# Reply to comment on: Urinary sodium excretion and its association with blood pressure in Nigeria: A nationwide population survey

**DOI:** 10.1111/jch.14354

**Published:** 2021-09-12

**Authors:** Augustine N. Odili, Babangida S. Chori

**Affiliations:** ^1^ Circulatory Health Research Laboratory College of Health Sciences University of Abuja Abuja Nigeria

Dear Editor,

We thank Khitan and colleagues^1^ for their comments on our recent article.^2^ We discussed some of the issues raised in the limitations section of the article; however, we wish to offer further explanations. Khitan and colleagues were concerned about the interpretation of our findings in terms of reverse causality. We think that this is outside the scope of the research. The cross‐sectional design of our study was not intended to explore the causal link between sodium and hypertension since temporality of association is a strong criterion for causality. Cross‐sectional studies at best will generate hypothesis which should be tested further using appropriate study designs. We however agree with the assertion that salt intake may be affected by several factors including medical conditions such as hypertension and heart failure. We made effort to adjust for the pre‐existing medical conditions. We achieved this by enquiring from patients if they were on antihypertensive medications or had previously been advised by any health professional to reduce salt intake on account of one cardiovascular disease or the other. Despite adjusting for these factors (use of antihypertensive drug and reduction in salt intake), the association of salt intake with blood pressure remained weak and non‐significant.

Judging from the median urinary creatinine value we reported, they posed another question regarding the adequacy of the 24‐h urine collected by the participants. Though a very laborious exercise, estimation of sodium excretion in 24‐h urine, as corroborated by Khitan and colleagues, is the gold standard for assessing salt intake. Various methods including Para Aminobenzoic acid (PABA) recovery and urinary creatinine excretion have been used previously to assess the adequacy of 24‐h urine collection in various epidemiological surveys but all of them have their limitations.^3^ On account of these limitations, two previous multi‐country survey—INTERMAP^4^ and INTERSALT^5^ did not use any of these two methods. Rather, they employed strict protocols which entailed (i) starting and stopping urine collection in person, and (ii) asking participants to redo urine collection if they reported missing a few drops, or (iii) if the 24‐h urine volume is < 250 ml. In addition to the use of strict protocols and expunging participants whose urine volume was less than 300 ml; we did not include individuals whose urinary creatinine coefficient were below 5^th^ or above 95^th^ percentile of distribution in the final analysis. About 11% of patients with complete records were not included in the final analysis as a result of this.

As noted in our article^2^ the urinary creatinine value was lower than that reported in the literature.^6^ We are convinced that the poor nutrition status of individuals residing in both rural and urban areas included in our study was responsible for this. Although Khitan and colleagues have argued otherwise by referring to one study conducted among African Americans in the USA^7^ and two other studies^6^, ^8^ conducted among Blacks living in Africa. They opined that since African Americans enrolled in the CARDIA study had higher level of serum creatinine compared to their European counterparts,^7^ urinary creatinine of our population should be higher than reported. Comparing our population with the blacks of the CARDIA study may be inappropriate. The CARDIA study enrolled blacks with incomplete African ancestry (averaged at 72.5%) and high socio‐economic status (where more than 65% earn over 25 000 dollars per annum). Similarly, in contrast to our study, other two studies among native Africans included individuals living in the urban cities of Kinshasa^6^ or Yaounde,^8^ majority of whom are young people. Expectedly, serum/urine creatinine should be higher among these populations given their higher socio‐economic status and lower average age. This is further supported by the fact that the urine creatinine we reported was lower compared to that reported in Kinshasa, while the urine volume compares favorably between the two populations.

## SOURCES OF FUNDING

Welcome Trust Seed Grant in Science (200755/Z/16/Z).

## CONFLICT OF INTEREST

None of the authors declares a conflict of interest.
